# Depression Among Patients With Tuberculosis at a Directly Observed Treatment Short-Course (DOTS) Center in Rural Delhi

**DOI:** 10.7759/cureus.30827

**Published:** 2022-10-29

**Authors:** Bhushan Kamble, Sunita Dhaked, Gunjan Mahaur, Bhabani Prasad, Pradeep Kumar, Gireesh K Dhaked

**Affiliations:** 1 Community and Family Medicine, All India Institute of Medical Sciences, Bibinagar, IND; 2 Community Medicine, Government Medical College, Dungarpur, IND; 3 Community Medicine, North Delhi Municipal Corporation (DMC) Medical College and Hindurao Hospital, Delhi, IND; 4 Preventive Medicine, Military Hospital, Jammu, IND; 5 Psychiatry, Institute of Medical Sciences, Varanasi, IND; 6 Gastroenterology, Sawai Man Singh (SMS) Medical College, Jaipur, IND

**Keywords:** tuberculosis, psychiatric disorder, patient health questionnaire‑9, directly observed treatment short‑course, depression

## Abstract

Background

Psychiatric disorders, particularly depression is prevalent among patients with tuberculosis (TB) and affect their treatment compliance. Patients with tuberculosis can develop depression due to multiple factors like longer treatment duration, social stigma, lack of family support, etc. In this study, depression and its associated factors were examined among patients with tuberculosis enrolled in a directly observed treatment short-course (DOTS) center in North Delhi.

Methods

In this DOTS center-based, cross-sectional study, 320 patients with pulmonary and extra-pulmonary TB above 18 years old were included. Basic socio-demographic information was gathered using a Hindi questionnaire, and depression was identified using the patient health questionnaire-9. People who received a score of 10 or higher were deemed to have depression. The Statistical Package for Social Sciences (SPSS) version 21 (IBM Corp., Armonk, NY, USA) was used for data analysis. Analysis between depression and no-depression groups was done by the chi-square test and a p-value* *< 0.05 was considered statistically significant.

Results

The study involved 320 patients in all, 193 (60.3%) of whom were men. The median age was 38 years, and the interquartile range (IQR) was 24 to 52 years. Depression was found to be present in half of the patients. Patients with a higher proportion of depression were male, belonged to the middle or below socio-economic status, were currently unemployed and literate, had monthly family income less than 8000 rupees, weight below 45 kg, used alcohol and tobacco, and were undergoing intensive phase (IP) of TB treatment (p-value< 0.05). Depression was not found to be associated with age, site of TB, previous history of anti-tubercular treatment (ATT) intake, marital status, and family size.

Conclusion

Depression among patients with TB is common and affects half of the patients afflicted with it. When evaluating patients with TB, physicians and DOTS providers should have a high index of suspicion for depression.

## Introduction

The disease tuberculosis (TB) is a leading cause of death from a single infectious agent and a significant contributor. According to the Global TB Report 2020, over 10 million individuals worldwide contracted TB in 2019 and 1.4 million died as a result of it. India accounted for approximately 26% of all TB cases worldwide [1]. Out of the 26 lakh cases that occurred in 2019, almost 80,000 people died from TB [2]. More than 264 million individuals around the world suffer from depression, a prevalent mental condition. Consistent sadness and a lack of interest in formerly fulfilling or joyful activities are its defining traits. The major cause of disability in the world today is depression, which also significantly increases the burden of sickness on the planet [3]. A person's capacity to function and lead a fulfilling life can be significantly affected by the consequences of depression, which can be long-lasting or recurrent [3]. A patient with TB may eventually develop depression due to a number of circumstances, including the prolonged TB treatment regimen, the stigma the patient experiences as a result of the illness, and the absence of family support [4]. Treatment with cognitive therapy for patients with TB reported as depressed led to a lower percentage of defaulters and a higher rate of treatment completion [5]. The prevalence of depression and associated factors in patients with TB registered at a Directly Observed Treatment Short-course (DOTS) program at the Rural Health Training Centre (RHTC), Alipur in North Delhi, were assessed in the present study.

## Materials and methods

Study design

This cross-sectional study was conducted for six months from July 2020 to December 2020. It included TB patients aged 18 years or above undergoing ATT at the DOTS center at RHTC, Alipur in North Delhi. The study was approved by the Institutional Ethics Committee, North DMC Medical College and Hindu Rao Hospital (approval no.: IEC/NDMC/2020/09).

Inclusion and exclusion criteria

Patients aged 18 and above who consented to the study and undergoing ATT were included. Those having drug-resistant TB, and chronic co-morbidities such as diabetes, human immunodeficiency virus (HIV), chronic kidney disease, stroke, cancer, or cardiovascular disease (CVD), etc. were excluded.

Sample size determination and sampling procedure

The formula n = 4 pq/l2 was used to calculate the sample size, where p is the known prevalence of the disease, q is 1-p, and l is the error. As per Salodia et al., [4] taking the prevalence of depression at 23.6% and considering an absolute error of 5%, the sample size came out as 289. A non-response rate of 10% was taken and the final sample size was 317. And so in the round figure, the total number of patients included was 320. Enrolment of the patients was done using consecutive sampling until a sample size of 320 was achieved.

Study variables

Data was collected using a questionnaire that included questions about the fundamental socio-demographic profile, TB information, and the Hindi version of the patient health questionnaire-9 (PHQ-9) for the diagnosis of depression. The PHQ-9 is a nine-item self-report questionnaire that asks participants to rate how frequently they have experienced any of the listed issues over the course of the past two weeks. Each question carries a score between 0 and 3, yielding results between 0-27. Scores ranging from 0 to 4 denote minimal or no depression, 5 to 9 mild depression, 10 to 14 moderate depression, 15 to 19 moderately severe depression, and 20 to 27 severe depression. People who received a score of 10 or higher were considered to have depression [6].

Data collection procedure

Patients were interviewed at the DOTS center. Written informed consent was taken before the interview, after explaining the purpose of the study. The patients who gave consent were made to sit in a separate room and an interview was conducted using a semi-structured questionnaire for approximately 10 minutes. The interview was conducted in the local language.

Data analysis and ethical considerations

Data were entered into Microsoft Excel 2019 (Microsoft Corp., Redmond, WA, USA). The Statistical Package for Social Sciences (SPSS) version 21 (IBM Corp., Armonk, NY, USA), was used for data analysis. Associations between categorical variables were examined using the chi-square test. A p-value of 0.05 was considered statistically significant. Approval from the Institutional Ethics Committee was taken before starting the study. All patients with depression were referred to the psychiatrist for further evaluation and treatment was given if needed, counseling about compliance to TB treatment was also done. No physical harm was done as there was no invasive procedure. Confidentiality was maintained at all stages of the study.

## Results

Socio-demographic characteristics

The study involved a total of 320 patients, out of which half were males (193; 60.3%). The median age of the study participants was 38 years, with an interquartile range (IQR) of 24 to 52 years. Most of the patients were married (254, 79.4%). The median monthly income of the patients was ₹ 8,000 (IQR ₹ 4,250 to ₹ 10,000). Current tobacco users in any form were 96 (30.0%), alcohol users were 68 (21.3%), 74 (23.1%) belonged to lower/middle socio-economic status (SES), mean weight of the patients was 47.1 kg with SD 11.8 kg, the median family size was four (IQR three to five).

Disease profile of patients

Out of 320, 170 (53.1%) were clinically diagnosed and 150 (46.9%) were microbiologically confirmed to be patients with TB. Pulmonary TB was found to be common (222, 69.4%) among the study participants as compared to extrapulmonary TB (98, 38.6 %). Most of the participants were new cases (284, 88.8%), and 36 (11.2%) had already undergone ATT in past. More than half the patients were in the continuation phase (CP) i.e., 201 (62.8%) and 119 (37.2%) were in the intensive phase (IP). Depression was present in 160 (50.0%) patients with TB (score more than 10 on the PHQ-9) and severe depression was found in 19 patients (PHQ-9 score: 20-27) (Table 1).

**Table 1:** Distribution of study participants as per the severity of depression (n=320) PHQ-9: Patient health questionnaire-9

Severity of depression	PHQ-9 score	N (%)
None/minimal	0-4	80 (25.0)
Mild	5-9	80 (25.0)
Moderate	10-14	80 (25.0)
Moderately severe	15-19	61 (19.06)
Severe	20-27	19 (5.94)

A higher proportion of depression was found in males who belonged to middle or lower socio-economic status, were currently unemployed and literate, had a monthly family income less than Rs. 8000, weighed below 45 kg, currently using alcohol and tobacco, and undergoing IP phase of TB treatment (P < 0.05). No association was found with depression and age, site of TB, previous history of ATT intake, marital status, and family size. Depression was found equally prevalent in pulmonary and extrapulmonary TB patients and more prevalent in the intensive phase as compared to the continuation phase (Table 2).

**Table 2:** Distribution of study participants according to the determinants of depression (n=320) ATT: Anti-tubercular treatment, CBNAAT: Cartridge-based nucleic acid amplification test, H/o: History of

S. No.	Determinants		Depression, N (%)			Total, N (%)		P-Value	
			Absent	Present					
Socio-demographic and basic characteristics:									
1	Age	18-44 years	97 (48.7)	102 (51.3)			199 (100)	0.564	
		≥ 45 years	63 (52.1)	58 (47.9)			121 (100)		
2	Gender	Male	86 (44.6)	107 (55.4)			193 (100)	0.016	
		Female	74 (58.3)	53 (41.7)			127 (100)		
3	Family size	< 4	96 (53.3)	84 (46.7)			180 (100)	0.176	
		≥ 4	64 (45.7)	76 (54.3)			140 (100)		
4	Weight of patient	< 45 kg	71 (47.3)	79 (52.7)			150 (100)	0.370	
		≥ 45 kg	89 (52.4)	81 (47.6)			170 (100)		
5	Employment status	Currently employed	95 (61.7)	59 (38.3)			154 (100)	0.00001	
		Currently unemployed	65 (39.2)	101 (60.8)			166 (100)		
6	Literacy	Illiterate	89 (57.1)	67 (42.9)			156 (100)	0.014	
		Literate	71 (43.3)	93 (56.7)			164 (100)		
7	Monthly family income	< 8000 ₹	83 (43.7)	107 (56.3)			190 (100)	0.006	
		≥ 8000 ₹	77 (59.2)	53 (40.8)			130 (100)		
8	Marital status	Married	125 (49.2)	129 (50.8)			254 (100)	0.581	
		Unmarried	35 (53.0)	31 (47.0)			66 (100)		
9	Socio-economic status [7]	Middle/lower	46 (62.2)	28 (37.8)			74 (100)	0.017	
		Upper/upper middle	114 (46.3)	132 (53.7)			246 (100)		
Disease characteristics and associated factors:									
10	Site of disease	Extrapulmonary	49 (50.0)	49 (50.0)	98 (100)			1.0	
		Pulmonary	111 (50.0)	111(50.0)	222 (100)				
11	Type of patients	New	133 (49.4)	136(50.6)	269 (100)			0.647	
		Old	27 (52.9)	24 (47.1)	51 (100)				
12	Case definition	Microbiologically confirmed (sputum/ CBNAAT)	107 (54.0)	91 (46.0)	198 (100)			0.066	
		Clinically confirmed	53 (43.4)	69 (56.6)	122 (100)				
13	H/o previous ATT intake	Yes	16 (44.4)	20 (55.6)	36 (100)			0.479	
		No	144 (50.7)	140(49.3)	284 (100)				
14	H/o alcohol intake	Yes	23 (33.8)	45 (66.2)	68 (100)			0.003	
		No	137 (54.4)	115(45.6)	252 (100)				
15	H/o tobacco use	Yes	31 (32.3)	65 (67.7)	96 (100)			0.00001	
		No	129 (57.6)	95 (42.4)	224 (100)				
16	Current treatment phase	Intensive phase	52 (43.7)	67 (56.3)	119 (100)			0.083	
		Continuation phase	108 (53.7)	93 (46.3)	201 (100)				

## Discussion

Studies examining the prevalence of depression among patients with TB globally were quite rare [8]. In our research, we discovered that 50% of patients with TB were depressed. The findings were similar to those of a cross-sectional study conducted by Dasa et al. in Eastern Ethiopia and a study undertaken by Dahiya et al. in Haryana, India [8,9]. It's also similar to other research done in Sub-Saharan Africa: Angola has 49.4% and the Southwest Region of Cameroon has a 61.1% prevalence of depression in patients with TB [10,11].

In comparison to other similar studies conducted worldwide, the prevalence of depression in patients with TB was higher in our study [4, 12-14]. The prevalence of depression estimated by various authors, varies significantly by study setting (rural vs. urban), as well as the screening instrument used [4]. A study conducted by Mandaknalli et al. revealed a 41.5 % depression prevalence among patients with TB using the PHQ-9, while Sulehri et al. found an 80% prevalence using the Beck depression inventory‑II [15,16].

Depression in TB can be mild and may not require any medical therapy or, it can be severe and necessitate a complete assessment and proper management [8]. Half of the patients with TB in this study had mild to moderate depression, while 5.94 % had a severe form of the disease that required therapy. These findings were comparable to those of Dasa et al. [8], who found mild to moderate depression in about half the patients. However, the frequency of severe depression was lower in this study than in ours, which could be attributed to gross geographical heterogeneity. Patients with a higher degree of depression may be less likely to adhere to anti-TB medications and increase the chances of drug resistance [17,18], lower quality of life and greater disability [17], lack anti-TB treatment adherence, and poor treatment outcomes, including death [8]. In our study, depression was found to be more prevalent among those who were male, belonged to middle or lower socio-economic status, and were unemployed. Similar findings have been reported by Salodia et al. [4]. Depression in patients with TB may be influenced by financial hardships. Both Sulehri et al. and Umang et al. have reported findings similar to our study [4,16].

Age, family size, or marital status did not appear to be associated with depression. According to our statistics, male patients with TB had a higher likelihood of becoming depressed. Being married does not offer any protection or benefits to patients with TB and depression. On the other hand, other research have revealed that depression in TB patients may be influenced by old age, substantial pathology, being single, and a lack of social support [9,19].

Strengths

One of the study's advantages is the use of a standardized and approved instrument (PHQ-9 in Hindi) for evaluating depression in patients with TB. Any possibility of investigator bias was minimized because the study was carried out by a single investigator. In our study, both the research question and objectives of the study were clearly stated. The sample size and study materials were collected scientifically.

Limitations

Although the sample size was calculated scientifically, the results of the current study cannot be generalized to all patients with TB in Delhi's rural areas because it was only undertaken at one DOTS location. The cross-sectional design of the study prevents making any conclusions about the temporal cause of depression. The cut-off score used to determine depression using the PHQ-9 scale may be another flaw in our study. To confirm the initial score and determine if participants who had a score of 5 to 9 had mild depression, a second delivery of the study tool was necessary. Due to the ambiguity of their initial scores, we decided not to include these people in the analysis because doing so might have significantly inflated the rate of depression among patients with tuberculosis.

## Conclusions

Half of the patients with TB in this study are reported to have a depressive disorder, indicating a significant frequency of depression among this population. A high index of suspicion should be made to check depression while assessing patients with TB by physicians and DOTS providers. People with lower socioeconomic status and those who are unemployed at the time of their sickness are more likely to suffer from depression. The family's financial problems may be made worse by their unemployment, which puts them at risk for depression. All patients with TB are therefore urged to undergo a psychiatric evaluation at least once while undergoing treatment and those who require it should have access to proper counseling and care. Prospective studies are needed to use repeated measures to monitor the development of depressive symptoms in patients with TB over time, starting on the day of the diagnosis itself, to identify the best time point during treatment to detect depression.

Recommendations

We recommend that whenever a patient with TB comes in contact with physicians and DOTS providers, suspicion should be maintained to check depression. If a patient shows any signs of depression, they should be sent to a counselor or psychiatrist. The DOTS center staff and caregivers should be trained about depression symptoms.
